# PD-1 Blockade Modulates Functional Activities of Exhausted-Like T Cell in Patients With Cutaneous Leishmaniasis

**DOI:** 10.3389/fimmu.2021.632667

**Published:** 2021-03-09

**Authors:** Renan Garcia de Moura, Luciana Polaco Covre, Carlos Henrique Fantecelle, Vitor Alejandro Torres Gajardo, Carla Baroni Cunha, Lorenzzo Lyrio Stringari, Ashton Trey Belew, Camila Batista Daniel, Sandra Ventorin Von Zeidler, Carlos Eduardo Tadokoro, Herbert Leonel de Matos Guedes, Raphael Lubiana Zanotti, David Mosser, Aloisio Falqueto, Arne N. Akbar, Daniel Claudio Oliveira Gomes

**Affiliations:** ^1^ Núcleo de Doenças Infecciosas, Universidade Federal do Espírito Santo, Vitoria, Brazil; ^2^ Division of Medicine, University College London, London, United Kingdom; ^3^ Department of Cell Biology and Molecular Genetics, University of Maryland, College Park, MD, United States; ^4^ Center for Bioinformatics and Computational Biology, University of Maryland, College Park, MD, United States; ^5^ Núcleo de Biotecnologia, Universidade Federal do Espírito Santo, Vitoria, Brazil; ^6^ Universidade Vila Velha, Vila Velha, Brazil; ^7^ Instituto de Biofísica Carlos Chagas Filho, Universidade Federal do Rio de Janeiro, Rio de Janeiro, Brazil; ^8^ Instituto Oswaldo Cruz, Fundação Oswaldo Cruz, Rio de Janeiro, Brazil; ^9^ Secretaria Estadual de Saúde do Espírito Santo-SESA, Vitoria, Brazil; ^10^ Departamento de Medicina Social, Universidade Federal do Espírito Santo, Vitoria, Brazil

**Keywords:** cutaneous leishmaniasis, *Leishmania braziliensis*, T cell exhaustion, PD-1, inhibitory checkpoint receptors, senescent T cells, immunosenescence

## Abstract

Patients infected by *Leishmania braziliensis* develop debilitating skin lesions. The role of inhibitory checkpoint receptors (ICRs) that induce T cell exhaustion during this disease is not known. Transcriptional profiling identified increased expression of ICRs including PD-1, PDL-1, PDL-2, TIM-3, and CTLA-4 in skin lesions of patients that was confirmed by immunohistology where there was increased expression of PD-1, TIM-3, and CTLA-4 in both CD4^+^ and CD8^+^ T cell subsets. Moreover, PDL-1/PDL-2 ligands were increased on skin macrophages compared to healthy controls. The proportions PD1^+^, but not TIM-3 or CTLA-4 expressing T cells in the circulation were positively correlated with those in the lesions of the same patients, suggesting that PD-1 may regulate T cell function equally in both compartments. Blocking PD-1 signaling in circulating T cells enhanced their proliferative capacity and IFN-γ production, but not TNF-α secretion in response to *L. braziliensis* recall antigen challenge *in vitro*. While we previously showed a significant correlation between the accumulation of senescent CD8^+^CD45RA^+^CD27^-^ T cells in the circulation and skin lesion size in the patients, there was no such correlation between the extent of PD-1 expression by circulating on T cells and the magnitude of skin lesions suggesting that exhausted-like T cells may not contribute to the cutaneous immunopathology. Nevertheless, we identified exhausted-like T cells in both skin lesions and in the blood. Targeting this population by PD-1 blockade may improve T cell function and thus accelerate parasite clearance that would reduce the cutaneous pathology in cutaneous leishmaniasis.

## Introduction

Leishmaniasis is caused by intracellular parasites belonging to *Leishmania* genus and has a global estimated prevalence of 12 million infected people, with 2 million new cases reported annually worldwide (1)*. Leishmania braziliensis* is the most prevalent of the cutaneous species in Brazil, causing chronic infections and skin tissue damage associated with a wide spectrum of clinical manifestations (2, 3).

The activity of antigen-specific T cells plays a central role in the clinical outcome of the disease, where nature, characteristics and profile of cytokines produced may influence the healing process or disease progression (4, 5). It is well recognized that interferon gamma (IFN-γ)-producing T cells are essential for mediating the leishmanicidal mechanisms and disease resolution. In contrast, increased production of TNF-α and non-specific cytotoxic mechanisms are linked to skin inflammation and lesion pathology (6, 7). Moreover, the absence of inhibitory mechanisms also has been correlated with tissue damage and severity of CL (8). Therefore, both insufficient and hyperactive non-specific immune responses may lead to pathology and provide avenues for therapeutic intervention.

T cells are subdued to avoid tissue damage during prolonged antigen exposure or chronic inflammation, progressively losing individual effector capabilities. This is regulated by the expression of inhibitory checkpoint receptors (iCRs) such as programmed death 1 (PD-1), T cell immunoglobulin-3 (TIM-3); Cytotoxic T-lymphocyte-associated protein 4 (CTLA-4); Lymphocyte activation gene-3 (LAG-3); and T cell immunoglobulin and ITIM domain (TIGIT) (9–11).

Evidence has highlighted a potentially deleterious role of IRCs during leishmania parasite infection (12–17). In this scenario, both CTLA-4 and PD-1 are highly expressed by CD8^+^ T cells from patients with visceral (13, 18) and diffuse cutaneous leishmaniasis (19). Similar, exhausted T cells expressing PD-1, TIM-3, 2B4, and CTLA-4 receptors are found on post-Kala-azar dermal (PKDL) and cutaneous leishmaniasis caused by *L. panamensis*, linked with the severity of the diseases (16, 20). In complement to these studies, the ICR blockade, particularly PD-1, has shown promise for augmenting specific T cell immunity in chronic inflammatory state, infectious diseases and cancer (9, 21–24). Although this may inadvertently exacerbate deleterious pro-inflammatory responses, it may also improve specific IFN-γ-dependent immunity (25). It has been noted that blocking ICR may limit exaggerated pro-inflammatory responses (26) which may be very relevant in the context of cutaneous leishmaniasis. This is supported by data from mice and dogs showing that PD-1 blockade and its ligands reduce the parasite burden, restores T cell proliferation and IFN-γ production (14, 17, 18, 27, 28). Furthermore, the expression of inhibitory checkpoint receptors decreases in VL cured patients (13). This suggests a potentially deleterious role of ICRs during infection that is poorly understood in the context of cutaneous leishmaniasis.

In this study, we identified the lesional transcriptomic signature and expression pattern of ICRs on circulating and skin lesional T cells during infection by *L. braziliensis*. We hypothesized that that PD-1 blockade could re-establish the functional activity of Ag-specific exhausted T cells to promote anti-*Leishmania* immunity. We found that exhausted T cells are widely distributed in both blood and lesional skin compartments during CL and that their function is inhibited by the PD-1 receptor. Moreover, the number of circulating PD-1 expressing T cells does not correlate with skin lesion size, suggesting that they are not involved in the disease pathology.

Overall the data present here suggests that exhausted cells co-exist with senescent T cells in the circulation and skin of patients with cutaneous leishmaniasis. While senescent T cells but not exhausted populations may contribute to the skin lesions, the exhausted population contributes to decreased immunity to the pathogen. The inhibition of PD-1 signaling may improve the immune response to the parasite in these patients.

## Materials and Methods

### Study Subjects

Peripheral blood from 15 untreated patients with cutaneous leishmaniasis (CL) attended at the University Hospital (HUCAM) of Universidade Federal do Espírito Santo, Brazil, were investigated in this study. They consisted of eight males and seven females with illness duration ranging from 30 to 120 days, lesion sizes ranging from 200–550 mm^2^ and age of 37 ± 13.6 years. The diagnosis of CL was based on clinical and laboratory criteria and all patients in this study tested positive for the PCR/restriction fragment length polymorphism of *L. braziliensis* and reported no prior infections or treatments. The control group (HC) consisted of 15 healthy age (41.4 ± 11.9) and gender-matched individuals with no history of leishmaniasis. All participants (patients and healthy volunteers) had seronegative testing for HIV, HBV and HCV infections, and had no history of chemotherapy, radiotherapy or treatment with immunosuppressive medications within the last 6 months. The patient and control samples were obtained before the COVID-19 outbreak. Patients provided written informed consent, and study procedures were performed in accordance with the principles of the Declaration of Helsinki. This study was registered at HUCAM ethical committee reference number 735.274.

### PBMC Isolation, Cell Sorting, and Culture

PBMC from HC and CL patients were isolated by centrifuging whole blood through a Ficoll-Hypaque (GE Healthcare) gradient followed by hemocytometry to determine the absolute number of viable then cryopreserved. Cells from both controls and patients were thawed in RPMI complete medium supplemented with 10% of fetal calf serum. Viability and recovery were measured using trypan blue dye exclusion.

### Flow Cytometric Analysis

For phenotypic and functional analysis, at least 10^6^ cells were stained washed and subsequently stained at 4°C for 20 min with the combination of surface antibodies. For intracellular analysis of cytokine secretion, cells were cultured at 37°C in the presence of monensin (used according to the manufacturer’s indication, BioLegend) and brefeldin A (5 mg/ml) (Sigma-Aldrich), added for the last 6 and 4 h of stimulation, respectively. Then, cells were fixed and permeabilized using the Cytofix/Cytoperm kit (BD Pharmingen), stained with NIR viability stain (Invitrogen) followed by cytokine intracellular antibodies on ice for 30 min. Data from 50.000 events obtained within CD3^+^ cells were acquired in a Fortessa X-20 cytometer (BD Biosciences) and analysed using FlowJo software (Treestar). ICRs gates were based on pooled fluorescence minus one control samples and applied identically across all samples. Gate strategy and used antibodies are described in the **Supplementary Figure 1** and **Table 1**, respectively.

### PD-1 Blockade

PBMCs were thawed, resuspended in complete media and incubated overnight at 37°C. The following day, cells were incubated with 10 μg/ml each of anti-PD-L1 (29E.2A3.C6) and anti-PD-L2 (24F.10C12.G12, both from Biolegend) antibodies as described previously (24) prior cell activation with anti-CD3 (OKT3, 0.5 μg/ml, Biolegend) or *L. braziliensis* promastigote antigens (LbAg, 10 μg/ml). After activation, cells were culture for 72 h. 10 μg/ml each of IgG2a (Mg2a-53) and IgG2b (MPC-11) isotype controls (Abcam) were used as control.

### Cytokine Determination

Cell culture were stimulated with 10 μg/ml of *L. braziliensis* promastigote antigens (LbAg) or 0.5 μg/ml plate-coated anti-CD3 (OKT3) and 5 ng/ml rhIL-2, with or without anti-PDL1/2 or isotype control antibodies. Culture supernatants were collected at 72h for the measurement of IFN-γ, TNF-α, and IL-10 by Cytokine Bead Array (CBA) (BD Biosciences) according to the manufacturer’s protocol.

### Proliferation Assay

Stimulated PBMC were cultured in the presence of PD-1 blocking or isotype control antibodies and rhIL-2 for 72 h as previously described (24). The proliferation was accessed by intracellular staining for the cell cycle related nuclear antigen Ki67 Alexa Fluor 647 (BD Bioscience) that was performed with Foxp3 Staining Buffer Set (Miltenyi Biotec) and analyzed by flow cytometric analysis.

### RNA-Seq Analysis

The RNA-Seq data was obtained from a previous study (29), which is available at the Sequence Read Archive (www.ncbi.nlm.nih.gov). Data as accessed through project accession reference #PRJNA307599, where paired-end reads (100 bp) were obtained through Illumina HiSeq 1500 platform. Samples analysed consisted of skin biopsies from uninfected controls (from endemic areas) (n= 10) and skin biopsies collected from patients infected with *Leishmania braziliensis* (n= 25). Initially, samples were trimmed using Trimmomatic (v. 0.39) to remove sequence adapters and filter low-quality (threshold of 25) bases at the start or end of reads. Samples were then aligned to the human reference genome (hg38/GRCh38 release 99) obtained from ENSEMBL (https://www.ensembl.org/) using Salmon (v 0.12.0) using the selective alignment option (–validateMappings) and correction for GC content bias (–gcBias). Transcript abundance at the gene-level was then calculated using tximport to obtain the counts table. Non-expressed, weakly expressed and non-protein code genes were removed prior to subsequent analyses, resulting in a count table of 17.668 genes. DESeq2, a package from Bioconductor, was used to define differentially expressed genes (30), considering genes with Benjamini-Hochberg p-adjusted value less than 0.05 as significant. The Variance Stabilizing Transformation (vst), available in the DESeq2 package, was used for visualization and clustering purposes. The function plotPCA, also available on the DESeq2 package, was used for the Principal Component Analysis. The ComplexHeatmap package (31) ggplot2 package (32) were used to generate the heatmap and plots, respectively. The heatmap was constructed to show the relative gene expression, where the vst normalized values were mean-centered across samples.

### tSNE Analysis

*t*-SNE is a non-linear dimensionality reduction method that optimally places cells with similar expression levels near to each other and cells with dissimilar expression levels further apart. Unbiased representations of multi-parameter flow cytometry data were generated using the t-distributed stochastic neighbour embedding (tSNE) algorithm. The R package “Rtsne” available on CRAN (github.com/jkrijthe/Rtsne) was used to perform the Barnes Hut implementation of tSNE on flow cytometry data. FlowJo software was used to export events of interest (in fcs format) for each sample. After using the Bioconductor “flowCore” R package to import.fcs file data and the Logicle transform to scale the data similarly to that displayed in FlowJo. 10,000 events from each sample analyzed in parallel were merged and the relevant fluorescent parameters were used.

### Skin Biopsies

Punch biopsies (8 mm in diameter) from the border of lesional skin were obtained from cutaneous leishmaniasis patients. Control skin punch biopsy specimens from healthy volunteers were also obtained. Biopsy specimens were frozen in OCT compound (Sakura, Alphen aan den Riji, The Netherlands). Six-micrometer sections were longitudinally sectioned to expose all skin layers and placed in poly-L-lysine coated slides (Star Frost^®^). Tissues were then fixed in acetone and ethanol and stored in -80°C until use.

### Immunohistochemistry

Frozen sections from HC skin and CL lesions were blocked with 1% bovine serum albumin (BSA) solution and incubated with primary antibodies (described in **Supplementary Table 1**). Staining was detected using Novolink™ Polymer Detection System Kit (Leica, RE7150-K) according to the manufacturer instructions. Slides were then dehydrated with xylol and ethanol and mounted with Entellan^®^ (MERK, 107960).

### Immunofluorescence

Immunofluorescence staining was performed using optimal dilutions of primary antibodies for 18 h at 4°C, followed by an appropriate fluorochrome-conjugated secondary antibody (described in **Supplementary Table 1**) incubation and mounting with Flouroshield Mounting Medium with DAPI (ABCAM, ab104139). Three images of the papillary and reticular dermis were captured at 200x magnification using fluorescence microscopy (Leica DMi8, Wetzlar, Germany). The number of positively stained cells was manually counted using computer-assisted image analysis (National Institutes of Health Image Software ImageJ 1.52j; https://imagej.nih.gov/ij/) and expressed as a percentage.

### Statistics

GraphPad Prism (version 7) was used to perform statistical analysis. Data distribution was verified using the Shapiro-Wilk test. Statistical significance was evaluated using the paired Student t-test. Mann-Whitney with Welch correction or Kruskal-Wallis test was performed for all continuous and nonparametric variables. Differences were considered significant when p was <0.05.

## Results

### Transcriptional Profiling Indicates that Inhibitory Checkpoint Receptors and Ligands Are Enriched in *Leishmania braziliensis* Lesions

**Figure 1:**
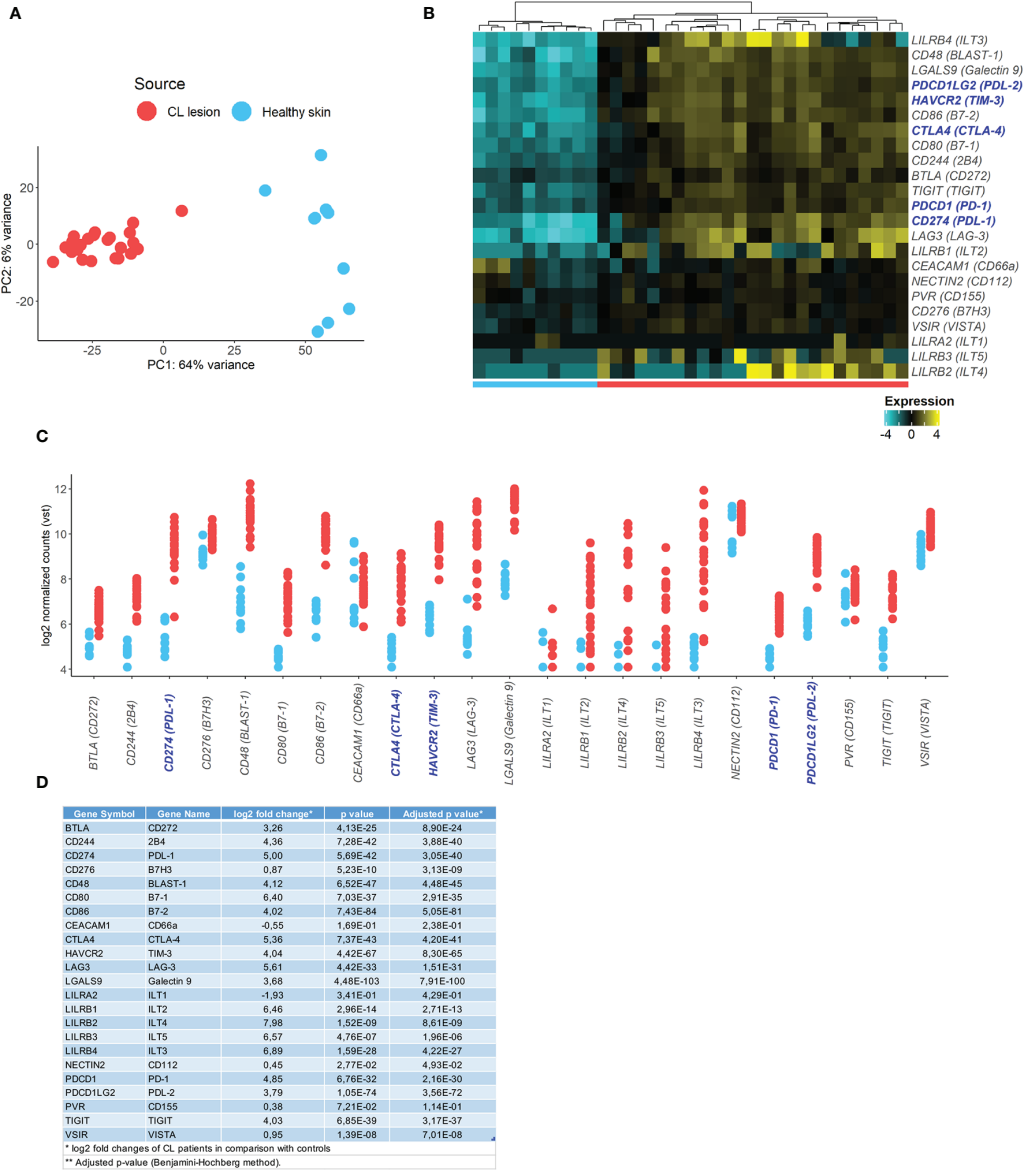
Identification of inhibitory checkpoint receptors gene expression signatures in CL lesions. **(A)** Principal components analysis showing principal component 1 (PC1) with variance of 64% and PC2 with variance of 6% of human transcriptome from 10 healthy volunteers (blue circle) and 25 CL patients (red circle). **(B)** Heatmap of exhaustion receptors and ligands genes expression. Columns represent individual healthy controls and CL patients and rows represent individual genes, colored to indicate relative expression levels (genes were mean centered across samples). **(C)** Plots showing expression of receptors [*PDCD1* (PD-1), *HAVCR2* (TIM-3), *CTLA4* (CTLA-4), *LAG3* (LAG-3), *TIGIT*, *CD244* (2B4), *NECTIN2* (CD112), *CD48* (BLAST-1), *PVR* (CD155), *TNFRSF14* (HVEM), *CD276* (B7H3), *CEACAM1* (CD66a), *LILRA2* (ILT1), *LILRB1* (ILT2), *LILRB4* (ILT3), *LILRB2* (ILT4), *LILRB3* (ILT5), *VSIR* (VISTA)] and ligands [*CD274* (PDL-1), *PDCD1LG2* (PDL-2), *CD80* (B7-1), *CD86* (B7-2), *LGALS9* (Galectin 9), *BTLA* (CD272), *VSIR* (VISTA)] in healthy skin (blue) and CL lesions (*red*). **(D)** Table with fold change and *p* -values of the analysed inhibitory checkpoint receptors lesional gene expression.

First, we analysed RNA-Seq data from a previous study where skin biopsies from uninfected controls were compared to biopsies collected from the border of lesions from patients infected with *Leishmania braziliensis* (29). We investigated transcriptional signatures for inhibitory receptors and their ligands in the skin during the active disease. Transcriptional profiles of skin from lesions were very different from healthy skin from control subjects, as demonstrated by principal component analysis (PCA) (**Figure 1A**). We found 9,955 genes identified as differentially expressed between the two groups that included inhibitory checkpoint receptors and their ligands that were uniquely associated with patient skin lesions (**Figures 1B, C**). Many genes associated with cellular exhaustion were increased in lesional skin and this included PD-1 (28.8 fold), TIM-3 (16.4 fold), CTLA-4 (40.9 fold), PDL-1 (32.0 fold), and PDL-2 (13.8 fold) (**Figures 1C, D**).

### Extensive Inflammatory Infiltrate with Increased Expression of Exhaustion-Related Receptors and Ligands in CL Lesions

**Figure 2:**
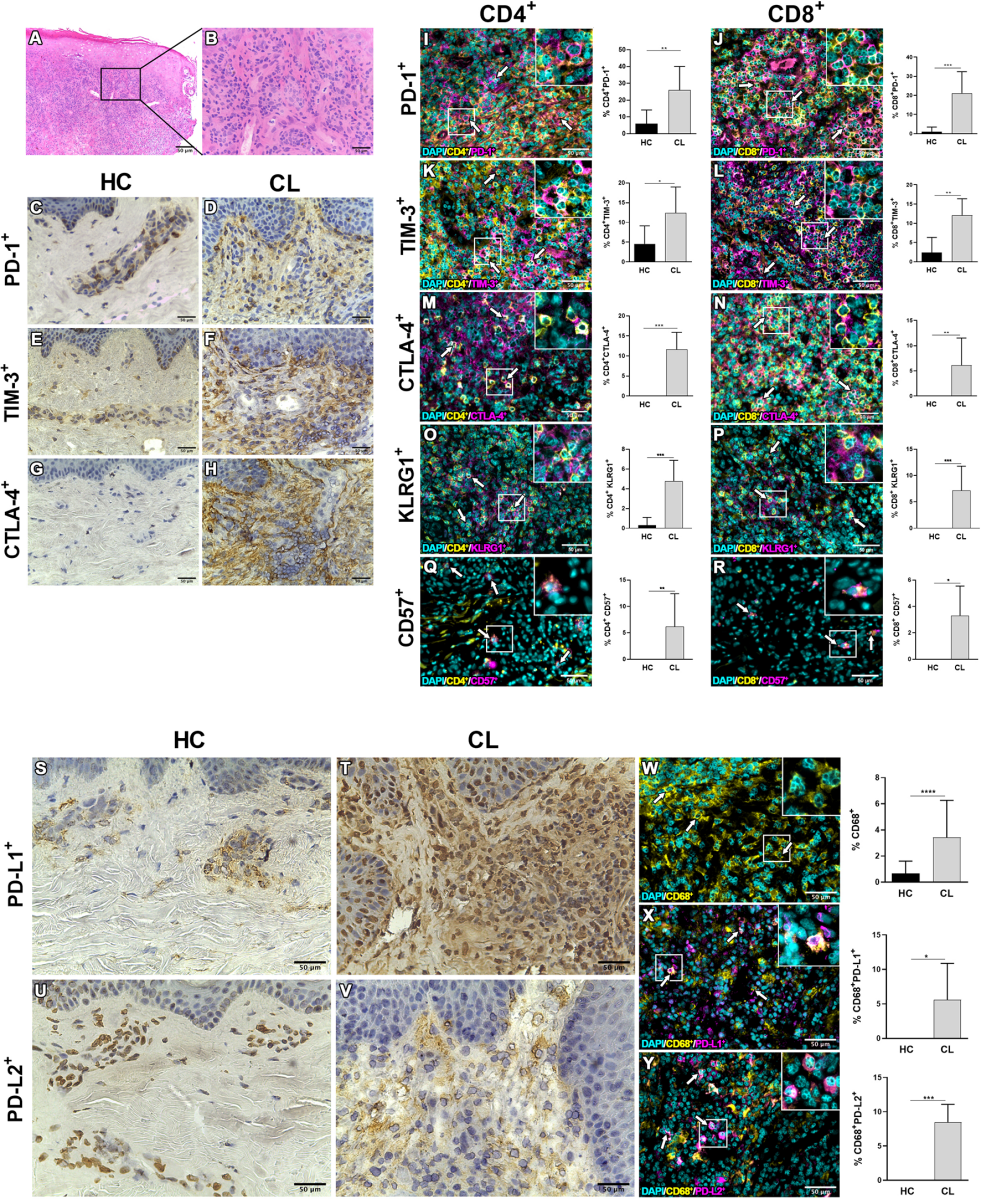
Inhibitory molecules and senescent-associated receptors are enriched in lesional skin during cutaneous leishmaniasis. **(A)** Representative hematoxylin and eosin staining of cutaneous leishmaniasis lesion with **(B)** dense inflammatory infiltrate. Immunohistochemistry staining (in brown) in healthy skin and CL lesions for PD-1 **(C, D)**, TIM-3 **(E, F)**, CTLA-4 **(G, H)**, PD-L1 **(S, T)**, and PD-L2 **(U, V)**. Immunofluorescence staining and cumulative data of the inhibitory checkpoint receptors PD-1, TIM-3, CTLA-4, and the senescence markers KLRG1 and CD57 expressed on CD4^+^ **(I, K, M, O, Q)** and CD8^+^ **(J, L, N, P, R)** cells from healthy controls (*n* = 8) and CL patients (*n* = 10). **(W–Y)** Representative staining and cumulative data of the expression of the inhibitory ligands PDL-1 or PDL-2 on dermal macrophages (CD68^+^) in healthy (*n* = 7) and lesional skin (*n* = 8). The white arrows indicate double-stained cells. The graphs show the mean ± SD. The *p*-values were calculated using Student’s *t* test with Welch’s correction or Mann-Whitney U-test. *p < 0.05, **p < 0.01, ***p < 0.001, ****p < 0.0001.

The expression of ICRs and senescence-associated receptors have been associated with several cell populations in inflammatory and non-inflammatory contexts. We next evaluated the expression of PD-1 and their ligands as well as TIM-3, CTLA-4, CD57, and KLRG1 (Killer cell lectin-like receptor G1) expression on lesional site. Histopathological analysis by H&E staining showed epidermal hyperplasia with a dense and diffuse inflammatory cell infiltrate involving the junction of the epidermis and dermis up to hypodermis, consisting mainly of lymphocytes, macrophages and plasma cells (**Figures 2A, B**). Immunohistochemical analysis revealed that this intense cell infiltration had increased expression of PD-1, TIM-3, and CTLA-4 compared to control skin (**Figures 2C–H**), supporting the RNAseq data. We also found that both CD4^+^ and CD8^+^ T cells were increased in the lesional inflammatory infiltrates compared to control skin (**Supplementary Figure 2A**). Furthermore, significantly greater proportions of these cells in the skin lesions of CL patients expressed PD-1, TIM-3, CTLA-4, CD57 and KLRG1 compared to the skin of healthy controls (**Figures 2I–R** and **Supplementary Figure 2B**). Moreover, there was increased expression of both PD-1 ligands (PDL-1 and PDL-2) (**Figures 2S–V**) that was found on lesional macrophages (CD68^+^) (**Figures 2W–Y** and **Supplementary Figure 2C**) and also neutrophils and fibroblasts (data not shown).

### Circulating T Cells of CL Patients Express Elevated Levels of Immune Checkpoint Receptors

**Figure 3:**
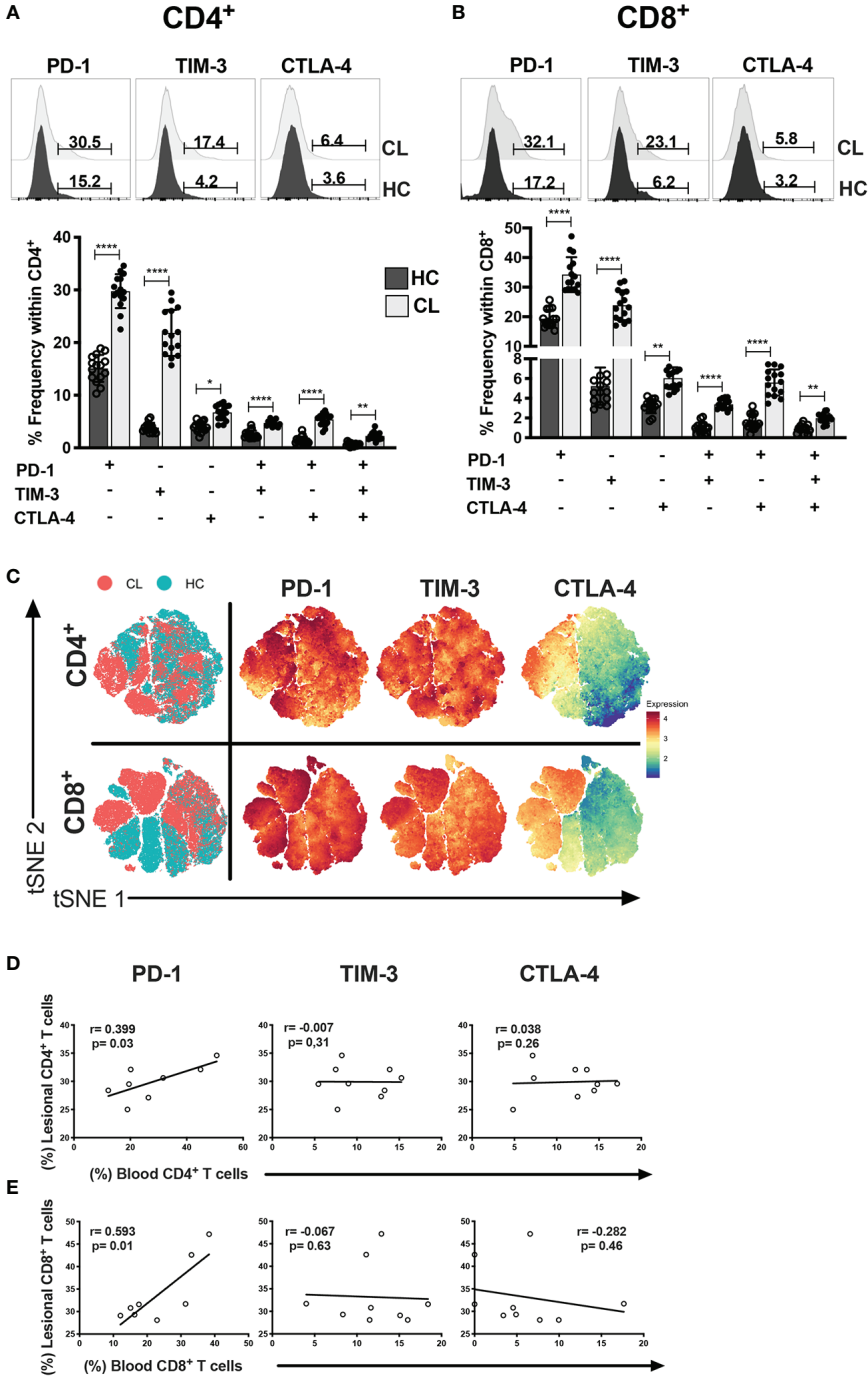
CL patients have multiple and single exhaustion receptor expression on circulating T cells. Representative histograms and cumulative data of percentage of PD-1, TIM-3 and CTLA-4 in CD4+ **(A)** and CD8+ **(B)** T cells isolated from healthy control- HC (n = 15) or patients with active cutaneous leishmaniasis-CL (n = 15). **(C)** tSNE performed gating on CD4+ and CD8+ cells from HC (blue dots) and CL (red dots) groups. The level of expression of PD-1, TIM-3, and CTLA-4 were evaluated separately on live cells generating the expression levels of the hierarchical clusters, represented in red for high expression, whereas blue represents low expression (cold-to-hot heat map). Scatterplot showing the Spearman’s correlation test relationship between frequencies of lesional and circulating **(D)** CD4+ and **(E)** CD8+ T cells expressing PD-1, TIM-3, or CTLA-4 (n = 10). The graphs show the mean with 95% of confidence. The p-values were calculated using Mann-Whitney test. *p < 0.05, **p < 0.01, ****p < 0.0001.

Circulating T cells are recruited to the skin during CL (33, 34). We next investigated the heterogeneity of ICRs on circulating T cell compartments. Both CD4^+^ and CD8^+^ T cells from CL patients exhibited elevated frequencies of cells that expressed PD-1, TIM-3, and CTLA-4 individually or combined (**Figures 3A, B**). We confirmed this by the tSNE algorithm that arbitrarily identified two different clusters of healthy control (Green) and cutaneous leishmaniasis patients (Red) groups (**Figure 3C**). In addition, the expression intensities and distribution of markers in each cluster were remarkably associated with both CD4^+^ and CD8^+^ T cells in CL patients.

Taken together these data suggest that T cells of CL patients have increased expression of ICRs that is not observed in healthy individuals. Thus, we next investigated whether there was an association between the level of individual ICRS on T cells in the circulation and in the skin lesions in individuals. We found a strong correlation between PD-1 expression in both compartments that was not observed with either TIM-3 or CTLA-4 (**Figures 3D, E**). This suggested that PD-1 may regulate T cell activity in both tissue compartments of CL patients to the same extent.

### Blocking PD-1 Enhances Leishmania-Specific Functions of T Cells from CL Patients

**Figure 4:**
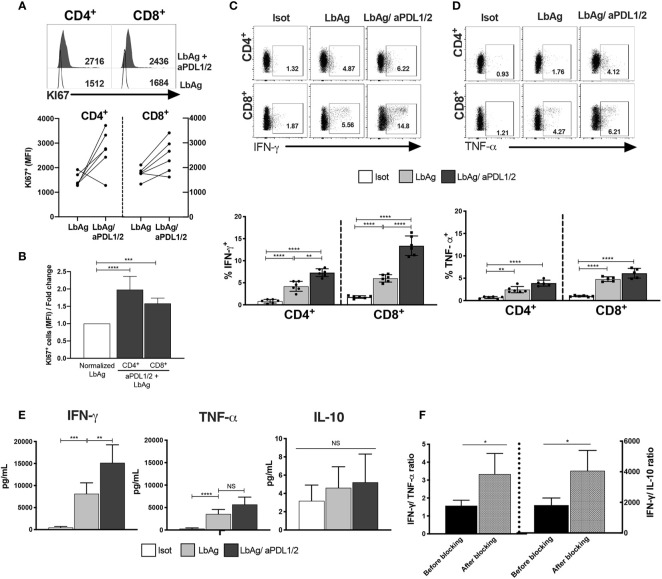
Proliferative and pro-inflammatory cytokines are increased by blocking PD-1 pathway in CL CD4^+^ and CD8^+^ T cells. **(A)** Representative histograms and pooled data showing Ki67 staining on CD4^+^ and CD8^+^ T cells from PBMC measured by flow cytometry after 72 h stimulation with 10 μg/ml of *L. braziliensis* promastigote antigens (LbAg). The cell cultures ware performed in the presence of 10 μg/ml anti-PDL1/2 antibodies. In control cultures, 10 μg/mL IgG2a, IgG2b were added (*n*= 6). **(B)** fold change of quantitative fluorescence intensity levels normalized with CD4^+^/CD8^+^ T cells stimulated with LbAg. **(C, D)** Representative dotplots and pooled data of frequencies of IFN-γ and TNF-α within CD4^+^ and CD8^+^ T cells after activation in the presence of PD-1 blocker. **(E)** Production of IFN-γ, TNF-α and IL-10 determined in the culture supernatants by CBA after activation with LbAg in the presence of PD-1 blockade. **(F)** Ratio between Ag-specific cytokines production before and after PD1 blockade. The graphs show the mean ± SEM. The *p*-values were calculated using Mann-Whitney test. *p < 0.05, **p < 0.01, ***p < 0.001, ****p < 0.0001, NS, not statistically significant.

PD-1- expressing T cells from CL patients are less responsive to both polyclonal and Ag-specific stimulation. We next investigated whether blockade of PD-1 signaling of circulating CD4^+^ and CD8^+^ T cells from CL patients, using antibodies to its ligands PDL-1 and PDL-2 blockade could enhance specific T cell functions linked to a leishmanicidal response after stimulation with *L. braziliensis* antigen (LbAg). The addition of anti- PDL1/2 significantly enhanced the proliferative capacity of CD4^+^ and CD8^+^ T cells, compared to cells that were activated in the absences of the blocking antibodies (**Figures 4A, B**). We also performed the same blocking experiment by stimulating T cells from both groups (HC and CL) with anti-CD3 and confirmed that blocking PD-1 signaling enhanced the proliferative capacity of T circulating cells from CL patients (**Supplementary Figure 3**).

We next investigated whether blocking PD-1 signaling affected cytokine production of CL patients after LbAg stimulation. We found that after blockade, the frequency of both IFN-*γ* TNF-α producing cells were significantly increased in CD4^+^ and CD8^+^ T cell compartments (**Figures 4C, D**). Interestingly, analysis of these cytokines in supernatants of cultures after LbAg stimulation in the presence or absence of PD-1 blockade demonstrated that only levels of IFN-*γ* production was significantly enhanced (**Figure 4E**). We also did not observe any effect of PD-1 blockade on the production of IL-10 in the same experiments (**Figure 4E**). The dominant cytokine response after PD-1 blockade was the enhancement of IFN-*γ* production and the ratio of IFN-*γ* to TNF-α and IFN-*γ* to IL-10 were both increased significantly (**Figure 4F**). As IFN-*γ* production has a role in protective immune responses while TNF-α secretion has an immunopathogenic consequences in this disease (5), the inhibition of PD-1 signaling would shift cytokine production towards immune protection in CL.

### PD-1 Expressing Cells Do Not Correlate with Lesion Size of CL Patients

**Figure 5:**
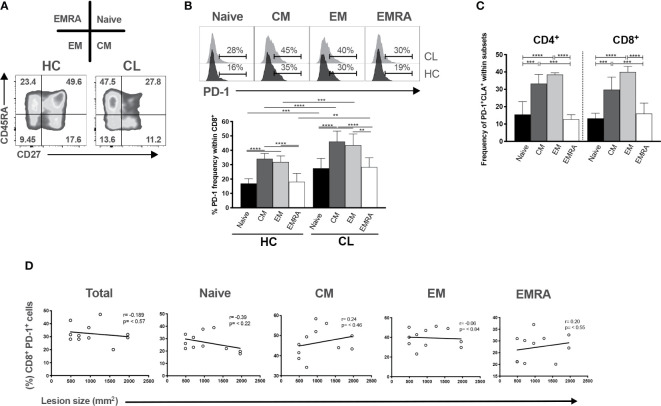
PD-1 expression is increased in differentiated CD8+ T cells but not correlated with lesion size of CL patients. **(A)** Representative plots of CD8+ T cells subsets isolated from HC and characterized by expressing CD45RA and CD27 markers (naïve-CD45RA+ CD27+; CM-central memory, CD45RA- CD27+; EM-effector memory, CD45RA-CD27-; and EMRA-effector memory T cells that re-express CD45RA, CD45RA+ CD27-). **(B)** Representative histogram and cumulative data of the ex vivo PD-1 frequencies within CD8+ subsets. **(C)** Pearson’s correlation test between frequencies of CD8+PD1+ T subsets (Naïve, CM, EM, and EMRA) and lesions size (mm^2^) of CL patients. **(D)** Cumulative data of the ex vivo PD1+ CLA+ within CD4+ and CD8+ subsets. The graphs show the mean ± SEM. The p-values were calculated using Mann-Whitney test. *p < 0.05, **p < 0.01, ***p < 0.001, ****p < 0.0001.

We showed previously that the size of skin lesions in CL correlated with the proportion of senescent CD8^+^CD45RA^+^CD27^-^ T cells in the circulation (34). We therefore investigated whether PD-1 expressing CD8^+^ T cells in peripheral blood were also associated with the cutaneous pathology in the patients. The relative expression of CD45RA and CD27 defined 4 different subsets of CD8^+^ T cells in both healthy controls and CL patients (**Figure 5A**). We investigated PD-1 expression within each of the 4 subsets and showed that PD-1 was significantly increased in all these population compared to healthy controls (**Figure 5B**). However, in both patients and controls, the senescent (CD45RA^+^CD27^-^) population expressed significantly less PD-1 compared to central memory (CM) or effector memory (EM) CD8^+^ T cells (**Figure 5B** bottom panel). In addition, we found increased frequencies of CM and EM subsets, but not senescent cells expressing PD-1 and CLA (**Figure 5C**). This suggest that a proportion of PD-1^+^ cells in the skin (**Figure 2**) may be derived from circulating populations. The proportions of total circulating CD4^+^PD1^+^ (data not shown) and CD8^+^PD1^+^ T cells and their subsets do not correlate with lesion size (**Figure 5D**). This suggests that despite the accumulation of PD1-expressing T cells in the circulation and in the skin, these cells may not be associated with the skin pathology that occurs in patients.

## Discussion

Immune checkpoint receptors regulate the magnitude of immune responses to protect against collateral tissue damage during immune responses to infection and to maintain peripheral self-tolerance. However their expression in chronic infection is associated with T cell exhaustion and pathogens persistence.

Here we identified a unique transcriptomic signature and expression pattern of these receptors on circulating and lesional T cells in patients with CL during infection by *L. braziliensis*. Previous studies have shown increased frequencies of ICRs on T cells during cutaneous leishmaniasis caused by *L. mexicana* (19) and *L. guyanensis* (16). Similarly, the increased expression of PD-1, PDL1 in patients with diffuse cutaneous leishmaniasis (DCL) or post-Kala-azar dermal leishmaniasis have been linked with a pronounced non-responsiveness of CTLs, diseases progression and parasite evasion (19, 20, 35). Conversely, it is well-recognized that CL late lesions contain few parasites, so the expression of ICRs as a mechanism that reduces immune activity and maintain parasite survival does not apply to *L. braziliensis* infection. Alternatively, it is possible that the chronic inflammatory state in the skin lesions promotes the expression of ICRs. This hypothesis is supported by accumulation of ICR expressing T cells during chronic inflammatory diseases such as Crohn’s, ulcerative colitis and rheumatoid arthritis, mainly maintained by TNF-α, IFN-*γ*, and IL-6, that are also seen in CL lesions (7, 8). In support of this, ICR- expressing cells have great ability to release a variety of inflammatory cytokines and mediate cytotoxicity, contributing to the progression of deleterious clinical outcomes (36–39). Therefore, the progression of tissue damage in leishmania infection would happen regardless of the ICRs expression.

CTLA-4 and PD-1 inhibit T cell function during *Leishmania* parasite infection (13, 16, 18, 20). Interestingly, the inhibition of PD-1 or CTLA-4 signaling on T cells is accompanied by an increase in proliferation and IFN-γ secretion that is correlated to better clinical status (14, 17, 18). Therefore, blocking ICRs could contribute to enhancing immunity against *L. braziliensis*. In our experiments the treatment with PD-1 blockade restored the proliferative capacity of T cells and preferentially augmented IFN-γ relative to TNF-α secretion in both CD4^+^ T and CD8^+^ T cell populations. While IFN-γ has been shown to have a protective role in CL, TNF-α secretion may promote pathology in the skin (33, 40, 41). Previous studies have shown that the control of inflammatory potential by blocking TNF-α *in vitro* or during antimonial therapy for CL *in vivo* are able to promote faster healing of lesions and higher cure rates than patients with anti-*Leishmania* treatment alone (42, 43). From our results we predict that this skewing of cytokine production may induce protective and non-pathogenic immunity since PD-1 blockade of CD8^+^ T cells does not exacerbate TNF-α secretion but instead induces protective IFN-γ based immune responses. Nevertheless, it will be important to ensure that checkpoint inhibitor inhibition does not inadvertently activate other inflammatory immune cells leading the exacerbation of CL

Our data also suggests for the first time the co-existence of senescent T cells and ICR-expressing (exhausted) T cells in the patients with CL. While the presence of senescent CD45RA^+^CD27^-^ (TEMRA) CD8^+^T cells in the circulation express CLA and are closely associated with skin lesion size (33), PD-1 expressing CD8^+^ T cells can also home to the skin but are not associated with lesion size. Senescent CD8^+^ T cells do not express high levels of PD-1 reinforcing the possibility that senescent and exhausted CD8+ T cells are distinct populations, as suggested previously (24).

Overall, the present study extends the understanding of local and systemic inhibitory checkpoint receptors expression patterns that occur in the context of *L. braziliensis* infection. Moreover, our data provide information about the compartmentalization of exhausted and senescent T cells in the blood and skin lesions of CL patients and provide a new rationale for therapeutic intervention against *Leishmania* infection.

## Data Availability Statement

The RNA-Seq datasets used in this study can be found in the online repository Sequence Read Archive (www.ncbi.nlm.nih.gov) through the project accession number PRJNA307599.

## Ethics Statement

The studies involving human participants were reviewed and approved by the Research and Ethics Committee of the Federal University of Espírito Santo, under reference number 735.274. The patients/participants provided their written informed consent to participate in this study.

## Author Contributions

LC, RG, CD, HM, SZ, VG, CC, and LS performed experiments. LC, DG, RG, CT, AB, and CF analyzed data. AF and RZ selected the patients. DG, AA, DM, and AF designed the project and discussed data. DG, AA, RG, LC, CF, and DM wrote the manuscript with the support from all other co-authors. All authors contributed to the article and approved the submitted version.

## Funding

This work was financially supported by the Fundação de Amparo a Pesquisa do Espírito Santo- FAPES/Newton Fund and Medical Research Council (Grant 72939273/16); the Fundação de Amparo a Pesquisa do Espírito Santo- FAPES (Grant 90/2017); the Fundação de Amparo a Pesquisa do Espírito Santo-FAPES/Ministério da Saúde (Grant 83152997/2018); and the Coordination for the Improvement of Higher Education Personnel - CAPES- Brazil and Medical Research Council (UK) (Grant MR/T015853/1).

## Conflict of Interest

The authors declare that the research was conducted in the absence of any commercial or financial relationships that could be construed as a potential conflict of interest.

The handling editor declared a shared affiliation, though no other collaboration, with one of the authors HM.

## Supplementary Material

Supplementary Figure 1 Representative gate strategy from healthy control donors and patients with cutaneous leishmaniasis.

Supplementary Figure 2
**(A)** Representative immunofluorescence staining and frequency of CD4^+^ and CD8^+^ cells (yellow) in healthy (*n* = 7) and lesional skin (*n* = 8). **(B)** Representative healthy skin sections stained for PD-1, TIM-3, CTLA-4, KLRG1 and CD57 (magenta) expression on CD4^+^ and CD8^+^ cells, and **(C)** PD-L1 and PD-L2 (magenta) on dermal macrophages (CD68^+^) (yellow). Nuclei (cyan) were stained with DAPI (ABCAM, ab104139). The graphs show the mean ± SD. The *p*-values were calculated using Kruskal-Wallis test. *****p* < 0.0001.

Supplementary Figure 3 Combined data showing Ki67 staining on CD4^+^ and CD8^+^ T cells from PBMC of heathy control donors (HC) and cutaneous leishmanisis patients (CL) measured by flow cytometry after 72 h stimulation with 0.5 μg/ml of anti-CD3. The cell culture was performed in the presence of 10 μg/mL anti-PDL1/2 antibodies. In control cultures, 10 μg/mL IgG2a, IgG2b were added. The *p*-values were calculated using Student’s *t* test with Welch’s correction or Mann-Whitney U-test. ***p* < 0.01, ****p* < 0.001, *****p* < 0.0001.

Supplementary Table 1 List of antibodies used for immunofluorescence, Immunohistochemistry and flow cytometry.
