# Supplementary figures and images for: PD-1 Blockade Modulates Functional Activities of Exhausted-Like T Cell in Patients With Cutaneous Leishmaniasis
Source: Front Immunol. 2021 Mar 9;12:632667. doi: 10.3389/fimmu.2021.632667 (PMC7985249; doi:10.3389/fimmu.2021.632667)

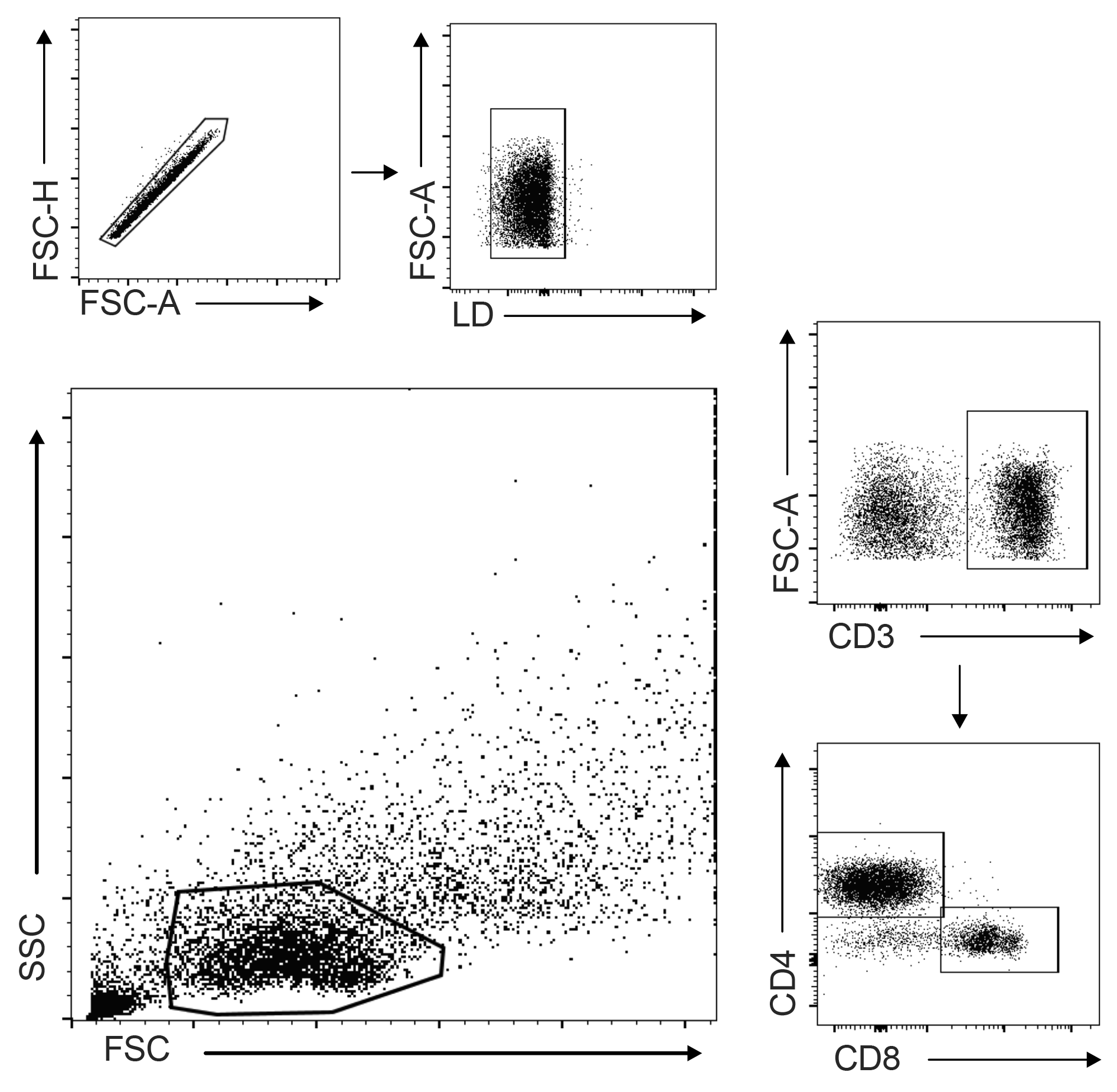

Supplement: Supplementary Figure 1 — Representative gate strategy from healthy control donors and patients with cutaneous leishmaniasis. [file Image_1.tif]

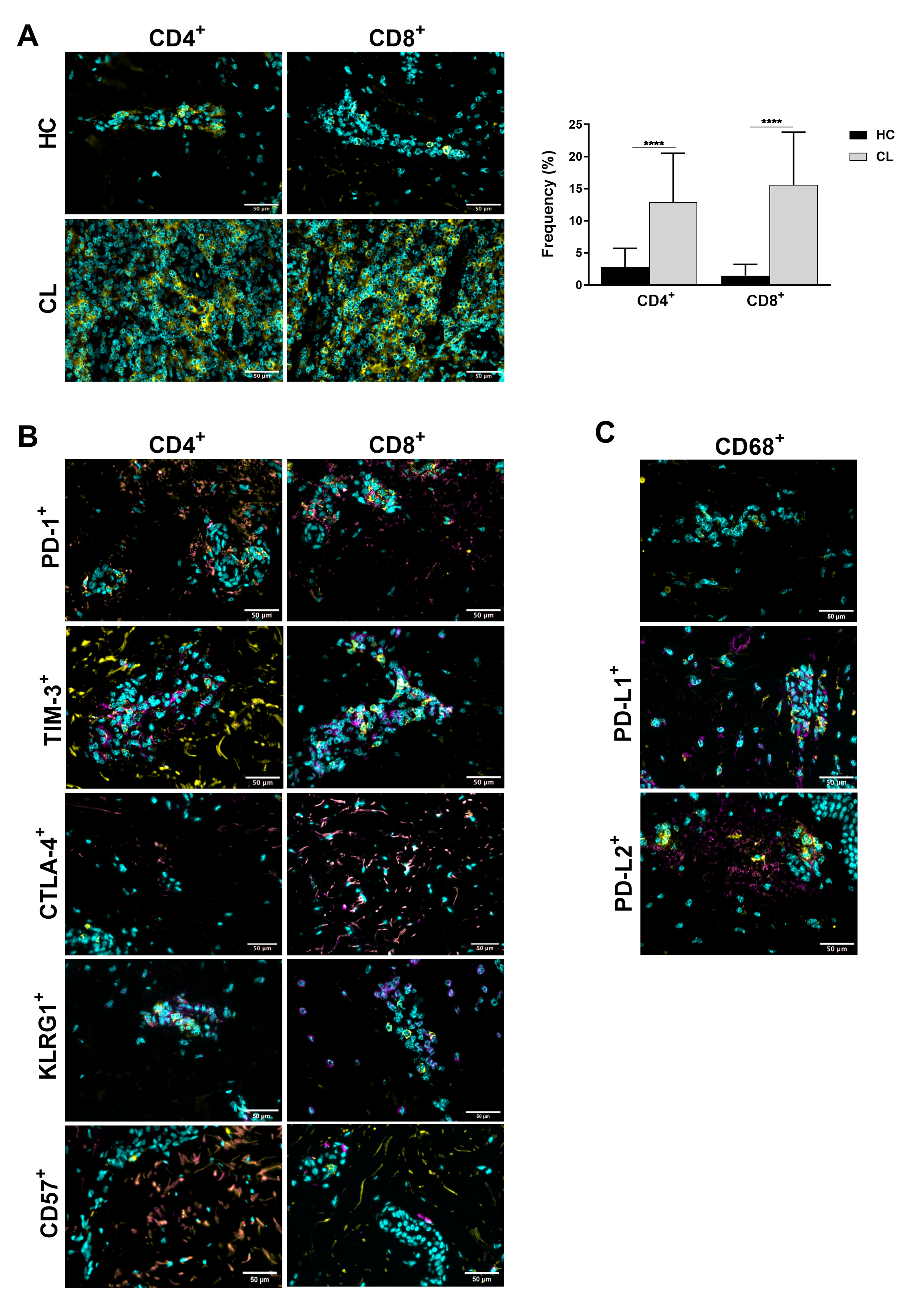

Supplement: Supplementary Figure 2 — (A) Representative immunofluorescence staining and frequency of CD4+ and CD8+ cells (yellow) in healthy (n = 7) and lesional skin (n = 8). (B) Representative healthy skin sections stained for PD-1, TIM-3, CTLA-4, KLRG1 and CD57 (magenta) expression on CD4+ and CD8+ cells, and (C) PD-L1 and PD-L2 (magenta) on dermal macrophages (CD68+) (yellow). Nuclei (cyan) were stained with DAPI (ABCAM, ab104139). The graphs show the mean ± SD. The p-values were calculated using Kruskal-Wallis test. ****p < 0.0001. [file Image_2.tif]

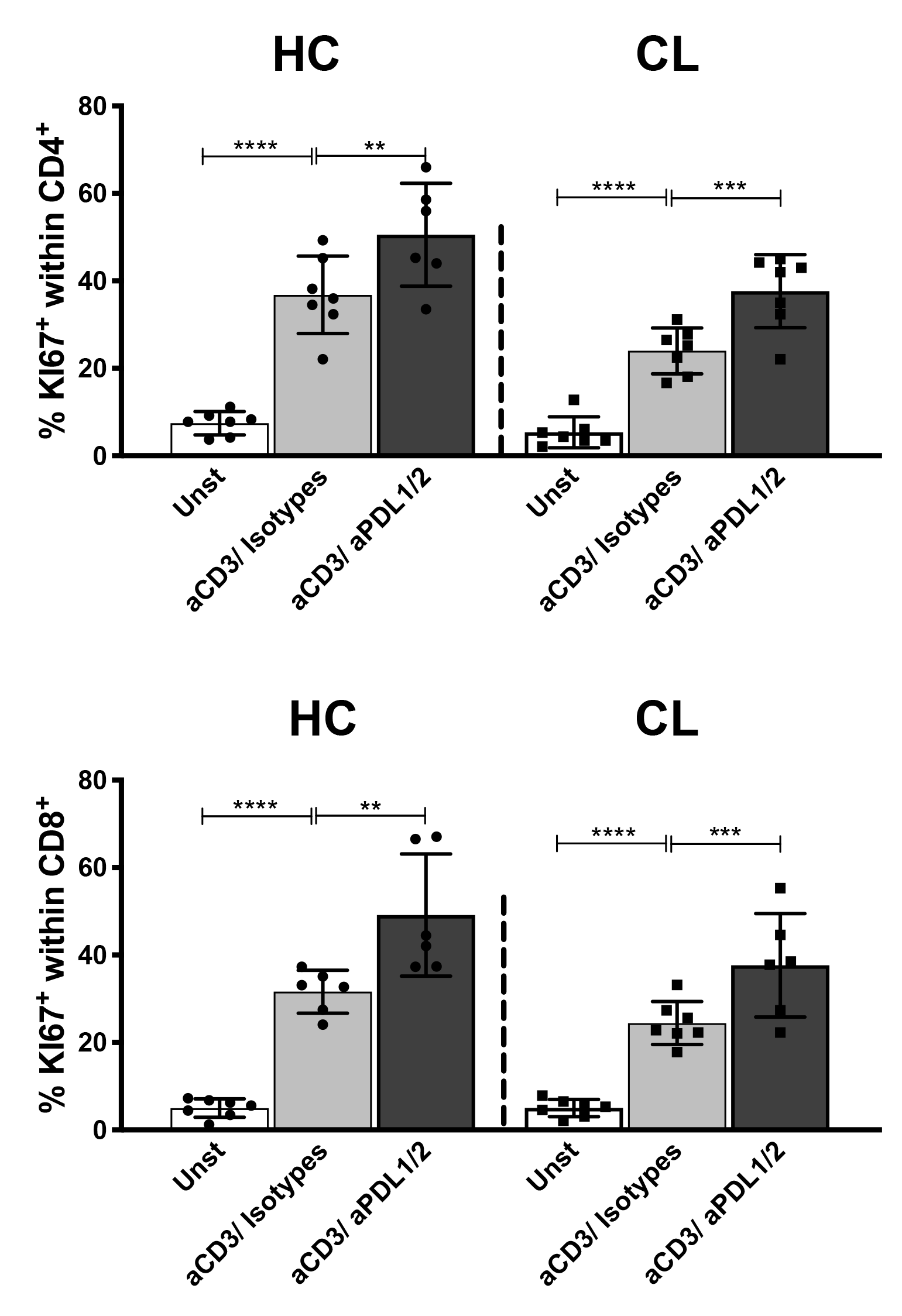

Supplement: Supplementary Figure 3 — Combined data showing Ki67 staining on CD4+ and CD8+ T cells from PBMC of heathy control donors (HC) and cutaneous leishmanisis patients (CL) measured by flow cytometry after 72 h stimulation with 0.5 μg/ml of anti-CD3. The cell culture was performed in the presence of 10 μg/mL anti-PDL1/2 antibodies. In control cultures, 10 μg/mL IgG2a, IgG2b were added. The p-values were calculated using Student’s t test with Welch’s correction or Mann-Whitney U-test. **p < 0.01, ***p < 0.001, ****p < 0.0001. [file Image_3.tif]
