# Supplementary material for: PD-1 Blockade Modulates Functional Activities of Exhausted-Like T Cell in Patients With Cutaneous Leishmaniasis
Source: Front Immunol. 2021 Mar 9;12:632667. doi: 10.3389/fimmu.2021.632667 (PMC7985249; doi:10.3389/fimmu.2021.632667)
Supplement: Supplementary Table 1 — List of antibodies used for immunofluorescence, Immunohistochemistry and flow cytometry. [file DataSheet_1.pdf]

Antibody	Clone	Isotype	Company
**immunohistochemistry and immunofluorescence**			
CD4	YNB46.1.8	IgG1	Bio-Rad
CD8	YTC182.20	IgG2b	Bio-Rad
PD-1	NAT105	IgG1	ab52587
TIM-3	Polyclonal	IgG	Abcam
CTLA-4	BNI3	IgG2a	BD Biosciences
CD68	Polyclonal	IgG	Abcam
PD-L1	ABM4E54	IgG2a	Abcam
PD-L2	MIH14	IgG1	Abcam
Goat anti-Rat	Polyclonal	IgG	ThermoFischer
Goat anti-Rabbit	Polyclonal	IgG	ThermoFischer
Goat anti-Rabbit	Polyclonal	IgG	ThermoFischer
Goat anti-Mouse	Polyclonal	IgG2a	ThermoFischer
Goat anti-Mouse	Polyclonal	IgG1	ThermoFischer
**Flow cytometry**			
Live/dead		Dead Blue	ThermoFischer
CD3	UCHT-1	PE-CF594	BD Biosciences
CD4	SK3	BV711	BD Biosciences
CD8	SK1	APC-H7	BD Biosciences
PD-1	EH12.1	PercP-Cy5.5	BD Biosciences
TIM-3	7D3	PE	BD Biosciences
CTLA-4	BNI3	BV605	Biolegend
TNF-α	MAb11	BV421	Biolegend
IFN-γ	4S.B3	APC	Biolegend
